# Impact of Epstein–Barr virus infection in patients with inflammatory bowel disease

**DOI:** 10.3389/fimmu.2022.1001055

**Published:** 2022-10-28

**Authors:** Hui Zhang, Shuliang Zhao, Zhijun Cao

**Affiliations:** Division of Gastroenterology and Hepatology, Key Laboratory of Gastroenterology and Hepatology, Ministry of Health, Shanghai Institute of Digestive Disease, Renji Hospital, School of Medicine, Shanghai Jiao Tong University, Shanghai, China

**Keywords:** inflammatory bowel disease, Epstein–Barr virus, viral colitis, lymphoproliferative diseases, immunosuppression

## Abstract

A high prevalence of Epstein–Barr virus (EBV) infection in patients with inflammatory bowel disease (IBD) has been reported in many case reports and studies; thus, the association between EBV and IBD has gained increasing attention. Patients with IBD are at an increased risk of opportunistic EBV infection owing to the common use of immunomodulators. EBV infection in IBD patients can cause various complications, including superimposed viral colitis, which is associated with chronicity, exacerbation, and poor prognosis of refractory IBD, and can induce progression to lymphoproliferative disorders, such as EBV-positive mucocutaneous ulcer (EBVMCU), lymphomatoid granulomatosis (LYG), hemophagocytic lymphohistiocytosis (HLH) and diffuse large B-cell lymphoma (DLBCL). It has been suggested to screen for EBV before initiating immunosuppressive therapy and monitor the status of EBV infection in patients with IBD, especially those who are EBV-seronegative and have a risk of primary EBV infection. Clinicians should also be careful of misdiagnosing IBD and EBV-associated lymphoproliferative diseases due to similarities in both clinical symptoms and endoscopic manifestations. Withdrawal of immunosuppressants has been shown to be an effective strategy to achieve remission of disease at the time of EBV diagnosis, but antiviral therapy remains controversial. The present review aims to describe the characteristics of the complications caused by EBV infection and generalize the recent research progress on and challenges caused by EBV infection in IBD patients. The literature for writing this review was collected from ‘PubMed’ research engine. The keywords ‘inflammatory bowel disease and Epstein–Barr virus’ or ‘ulcerative colitis and Epstein–Barr virus’ or ‘Crohn’s disease and Epstein–Barr virus’ were used to collect the literature and relevant papers were collected to help writing this review.

## Introduction

Inflammatory bowel disease (IBD) is a well-characterized syndrome that includes Crohn’s disease (CD), ulcerative colitis (UC), and inflammatory bowel disease unclassified (IBDU), which have closely related but heterogeneous disease processes and manifest with alternating periods of exacerbation and remission. The pathogenesis of IBD is still unclear; the current leading theory involves uncontrolled immune-mediated chronic inflammation in the intestinal mucosa of genetically predisposed individuals responding to an unknown environmental trigger that interacts with the intestinal gut flora (1).

Epstein–Barr virus (EBV) is a member of the herpesvirus family that successfully infects over 90% of people, mostly in childhood, with lifelong persistence in the latent phase in resting memory B cells. Primary EBV infection during childhood is usually asymptomatic, but when it occurs in adolescence or adulthood, most cases manifest clinically as infectious mononucleosis (IM), which is usually a self-limiting disease (2). However, EBV can be reactivated, especially in immunocompromised people whose immune systems are impaired, resulting in aggressive and even fatal lymphoproliferative diseases such as chronic active EBV infection (CAEBV), hemophagocytic lymphohistiocytosis (HLH), and B- or T/NK-cell lymphomas (2–4). Moreover, other malignant diseases, including nasopharyngeal carcinoma (NPC), post-transplant lymphoproliferative disease (PTLD), gastric adenocarcinoma, and autoimmune diseases, have been reported to be associated with EBV infection (5–8).

**Figure 1:**
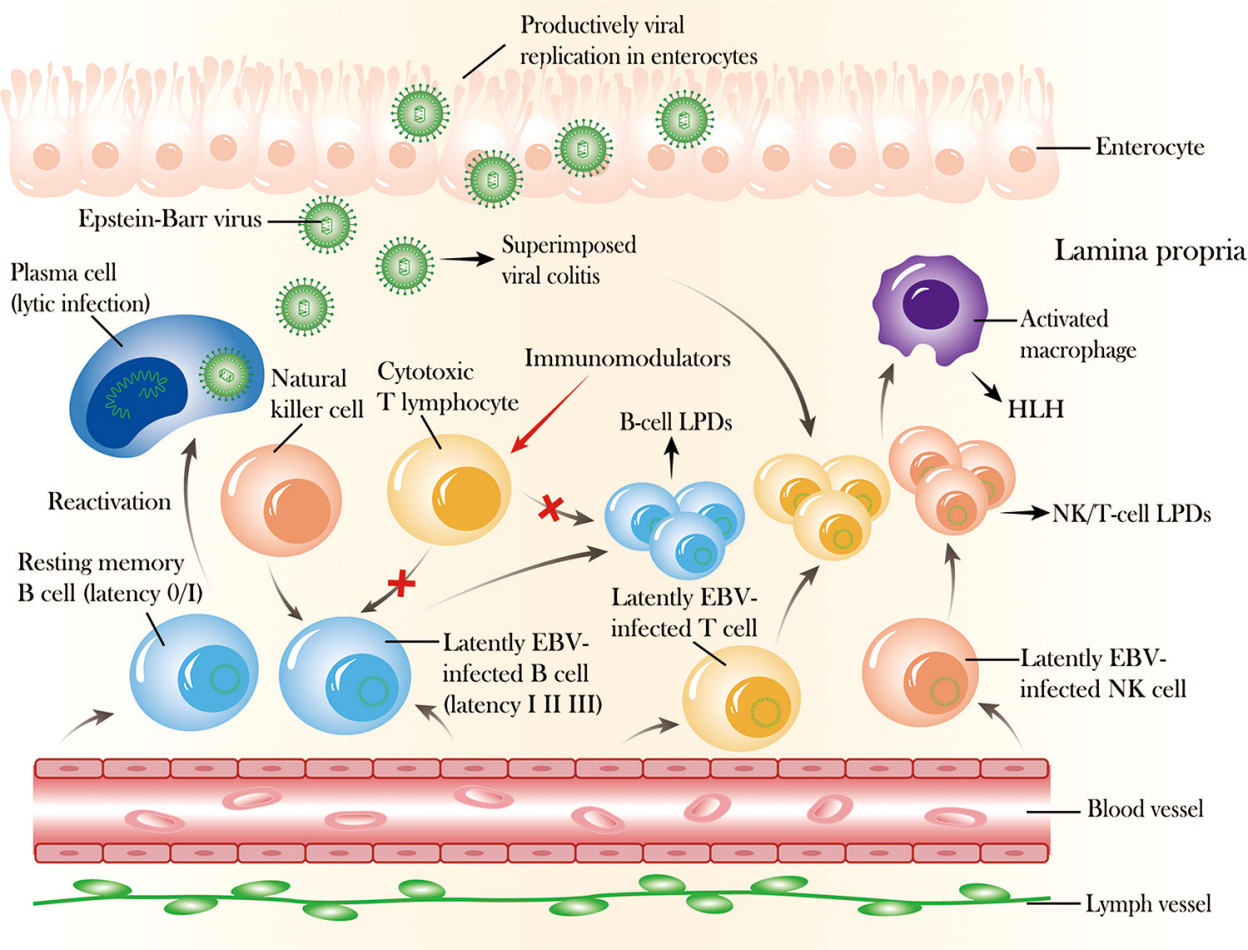
The mechanism and outcome of Epstein–Barr virus (EBV) reactivation in the intestinal mucosa. After primary infection, EBV can express different proteins (latency I, II, III) in latently infected B cells that can be recognized and controlled by cytotoxic T lymphocytes and natural killer (NK) cells, and EBV can exist permanently in resting memory B cells (latency 0/I) in healthy immunocompetent individuals. In addition, EBV can also infect NK or T cells, with prolonged latent infection. However, EBV can be reactivated from latency 0/I to cause lytic infection with active production of virus, and latently infected B or NK/T cells can undergo activation and proliferation due to EBV replication associated with the long-term use of immunosuppressive agents or biologics, which impairs cellular immunity, causing superimposed viral colitis and even B-cell or NK/T-cell lymphoproliferative diseases. Moreover, EBV infection can also spread from lymphocytes to enterocytes, where lytic replication occurs, destroying the mucosal tissue.

An increasing number of studies have verified EBV in the peripheral blood or the intestinal mucosa using the techniques of qPCR and *in situ* hybridization for EBV-encoded small RNA1 (EBER1) in IBD patients (9–11), and EBV infection may play a role in the exacerbation of the IBD clinical course, resulting in refractory IBD (12–14). In patients with IBD and opportunistic EBV infection, latent EBV can transform into lytic EBV, causing EBV-related colitis, lymphoproliferative diseases, and occasionally malignant lymphomas; this transformation is likely related to the long-term use of immunosuppressants or biologics and chronic inflammation itself (**Figure 1**) (10, 13, 15, 16). This review aims to describe the characteristics of the complications caused by EBV infection and generalize the recent research progress on and challenges associated with EBV infection in IBD patients.

## Evaluation of intestinal EBV infection in IBD patients

### EBV life cycle

During primary infection, EBV infects B lymphocytes in the oropharyngeal mucosa, and possibly, after an initial transient burst of replication in the oropharyngeal epithelium, latency is induced. In most immunocompetent individuals, EBV exists persistently in the latent phase in resting memory B cells and does not cause clinical symptoms because newly infected and differentiating B cells can be controlled by cytotoxic T-cell (CTL) responses. The EBV genome consists of a linear DNA molecule that encodes nearly 100 viral proteins that are expressed during replication, whereas only 10 are expressed in latently infected B cells, including two types of noncoding RNAs (EBER1 and EBER2), six nuclear proteins (EBNA1, EBNA2, EBNA3A, EBNA3B, EBNA3C and EBNA5) and two membrane proteins (LMP1 and LMP2), which help EBV escape virus-specific, cell-mediated immune responses of the host in immunocompetent individuals. There are four latency patterns (latency 0, I, II, III) of EBV gene expression, and three of them are observed in EBV-associated diseases. Latency I is associated with Burkitt’s lymphoma, and only EBNA-1 and EBER are expressed in this form. Latency II is associated with nasopharyngeal carcinoma, Hodgkin’s lymphoma, and peripheral T-cell lymphoma, and EBNA-1, LMP-1, LMP-2, and EBER are expressed in this second form. Latency III is associated with infectious mononucleosis and X-linked lymphoproliferative disease, and all the genes (EBNA-1, EBNA-2, EBNA-3, LMP-1, LMP-2, and EBER) are expressed in this third form (2). Moreover, EBV can be reactivated and shift to the lytic phase of infection in a subset of immunocompromised individuals, such as patients with cancers or autoimmune diseases, initiated by the immediate early transcription factors BZLF1 and BRLF1 and accompanied by the differentiation of B lymphocytes into plasma cells (17).

### The presence of EBV infection in IBD patients and cut-off point for distinguishing between nonpathogenic EBV latent infection and superimposed viral colitis

Many studies have confirmed the presence of EBV infection in mucosal inflammatory cells of patients with IBD (**Table 1**); thus, the relationship between EBV infection and IBD has gained increasing attention (9–11). Various techniques have been developed to diagnose EBV infection, such as blood analysis, EBV viral load (EBV-DNA) measurement by polymerase chain reaction (PCR), serological EBV-specific antibody testing, EBV gene product identification by immunohistochemistry (IHC), EBV-encoded small RNA (EBER) detection by *in situ* hybridization (ISH) and EBV DNA detection in biopsies of colon mucosa by PCR; among these techniques, EBER-ISH is considered the gold standard due to its high sensitivity and precise cellular localization (10, 11, 25). Although over 90% of the population has positive serology (2), performing serological testing to assess EBV infection has some limitations, as most patients with IBD are serologically IgG positive; however, this implies only prior infection. There are very few cases of IgM positivity, which indicates recent infection, easily leading to a false negative result in immunocompromised patients with IBD (13, 22). PCR is used to identify the presence of EBV in the intestinal samples of patients with IBD but cannot identify the exact location of EBV in the colonic tissue (12). The application of *in situ* hybridization for EBER in many studies helped identify EBV-infected cells, which were mainly B lymphocytes, in the colonic mucosa in both Western and Asian population (9, 10, 22). Immunohistochemical staining for EBV-specific lytic proteins BMRF1 and BZLF1, indicators of a switch from the EBV latent phase to the lytic phase (22, 25), was performed in some studies and verified the existence of EBV lytic infection in the colonic mucosa of patients with IBD (10, 16, 22). Ciccocioppo et al. used real-time PCR to investigate EBV localization and the viral DNA load in different cell populations in the colonic mucosa of IBD patients for the first time and discovered the presence of EBV DNA in enterocytes isolated from colonic mucosa. This study also found that the lack of ZEBRA (BZLF1) staining in those enterocytes carrying a high viral load cannot rule out the presence of the productive viral replication although it is now accepted that the role of epithelial cells in the EBV life cycle is to support the replication and spread of the virus within the host (16).

**Table 1:** Epstein–Barr virus-associated viral colitis in IBD patients and the influence of EBV on the clinical course of IBD.

Serial number	Samples	Techniques	Prevalence	Risk factors	Influence on the clinical course of IBD	Additional comments	References
1	surgically resected colonic specimens	ISH for EBER-1, IHC	62.5% in IBD (63.6% in CD, 60.0% in UC)	NA	NA	EBV-infected cells were mostly located in B lymphocytes and histiocyte-shaped cells	Hideo Yanai et al. (9)
2	colon tissue specimens	IHC, ISH for EBER and lgLC	71.4% in IBD (81.0% in CD, 60.0% in UC)	NA	influenced the composition of inflammatory infiltrate in UC and may contribute to self-perpetuation of the disease as well as the development of autoimmune disease	the presence of EBV lytic infection, EBER-positive cells were mainly B lymphocytes, higher frequencies of high EBV load and productive infection in active UC related to the characteristics of the TH2 patten	Spieker et al. (10)
3	colon tissue specimens	IHC, ISH for EBER and NotI/PstI, serology, PCR, southern blotting	41.0% in UC	NA	contributed to the chronicity of UC	the presence of linear viral DNA indicated EBV lytic infection, the immune escape of EBV infection mediated by the IL-10 molecule and activation of latently infected resting B cells caused by EBV-specific helper CD4+T-cells	Bertalot et al. (11)
4	whole blood and intestinal tissue samples	PCR	25.5% for blood and 46.8% for intestinal tissue in IBD	NA	contributed to the exacerbation of IBD	a higher prevalence of EBV DNA in intestinal samples in IBD patients than in controls, a disconnection between the levels of EBV load detected in peripheral blood and that in mucosal samples	Dimitroulia et al. (12)
5	intestinal biopsies and blood available undergoing EBV testing	ISH for EBER, PCR, histopathology and immunohistochemistry	48.3% in IBD	combinations of immunosuppressive drugs	increased surgery risk	EBER-positive cases were divided into two categories: low EBV concentration (< 10/HPF) and high EBV concentration (≥ 10/HPF), a high EBV load was correlated with monomorphic lesions, a disconnection between the levels of EBV load detected in peripheral blood and that in mucosal samples	Nissen et al. (18)
6	peripheral blood and intestinal samples	PCR, IHC	52.5% in IBD	use of biologic agents and topical steroids	associated with the severity of microscopic lesions and high endoscopic activity indexes in refractory IBD	a cut-off value of 103 copies/105 cells for EBV DNA in the colonic mucosa to distinguish between superimposed viral colitis and latent viral infection, a disconnection between the levels of EBV load detected in peripheral blood and that in mucosal samples	Ciccocioppo et al. (13)
7	colonic mucosal biopsies	PCR, IHC, ISH for EBER	NA	NA	associated with the severity of mucosal damage and clinical activity indexes	the presence of EBV DNA in enterocytes isolated from the colonic mucosa, opportunistic viral infection in inflamed IBD mucosa may spread from immune cells to epithelial cells	Ciccocioppo et al. (16)
8	colonic mucosal biopsies	PCR	15.8% in IBD (21.2% in CD, 9.3% in UC)	colonic mucosal inflammation and use of corticosteroids, CyA or TAC	associated with subsequent colectomy	a higher prevalence of EBV DNA in UC patients than in CD patients and healthy controls	Hosomi et al. (19)
9	colonic tissue	ISH for EBER-1	54.4% in IBD, 60.0% in refractory IBD	NA	associated with depth of inflammation and mucosal ulceration in refractory IBD	defined focal EBER-1 positivity as EBER-positive cells < 5/HPF and diffuse EBER-1 positivity as EBER-positive cells ≥ 5/HPF	Pezhouh et al. (14)
10	colonic mucosa	PCR, ISH for EBER	33.0% in IBD	NA	associated with clinical disease activities	a slight agreement of PCR and ISH for intestinal mucosa	Li et al. (20)
11	blood and intestinal mucosa	PCR, ISH for EBER	8.4% for blood samples, 56.3% in EBV DNA- positive IBD	older age, AZA/6-MP therapy, UC	linked to the aggravation of mucosal inflammation and refractoriness in IBD	EBER-positive cases were divided into two categories: low EBV concentration (< 10/HPF) and high EBV concentration (≥ 10/HPF), a disconnection between the levels of EBV load detected in peripheral blood and that in mucosal samples, EBV infection does not necessarily indicate poor prognosis	Zhou et al. (21)
12	peripheral blood and intestinal biopsies	PCR, ISH for EBER, IHC	39.1% for superimposed viral colitis in IBD	age, steroid dependence, and irregular ulceration	positively correlated with disease activity and adverse outcomes, including surgery rates, more hospital admissions and longer hospital stays	the presence of EBV lytic infection, the best EBER cut-off point for outcome prediction was 2.5/HPF and the cut-off value for blood EBV DNA was set to 2035 copies/mL, EBER-positive cells were mainly B lymphocytes	Xu et al. (22)
13	Peripheral blood and colonic mucosal samples	PCR	52.3% for blood samples and 79.4% for mucosal samples	clinical disease activity	related to clinical disease activities and contributed to acute exacerbation of IBD	the sensitivity of peripheral blood tests is too low to meet the needs of clinical surveillance of the virus, other non-invasive assays need to be further explored	Wang et al. (23)
14	intestinal biopsies	ISH for EBER	46% in IBD	active steroid treatment	associated with higher hospital admission and surgery rates and the need for escalation in therapy	EBV infection was associated with severe histological activity and presence of a lymphoplasmacytic infiltration	Núñez Ortiz et al. (24)

IBD, inflammatory bowel disease; UC, ulcerative colitis; CD, Crohn’s disease; EBV, Epstein–Barr virus; ISH, in situ hybridization; EBER, EBV-encoded small RNA; IHC, immunohistochemistry; PCR, polymerase chain reaction; CyA, cyclosporine; TAC, tacrolimus; AZA, azathioprine; 6-MP, 6-mercaptopurine; NA, not available.

Although many studies have confirmed the presence of EBV infection in peripheral blood or intestinal tissue of patients with IBD, few studies have attempted to distinguish between latent nonpathogenic EBV infection and superimposed viral colitis, and those that have tended to confuse the two conditions, leading to extremely different outcomes among IBD patients (9–12). Of course, immunohistochemical staining for BZLF1 can help confirm the existence of lytic EBV infection in the intestinal tissues of IBD patients, but BZLF1-positive cells have been detected in very few IBD cases, suggesting a limited value in differentiating lytic infection from latent infection (10, 22). Pezhouh et al. defined focal EBER-1 positivity as EBER-positive cells in < 5/high-power fields (HPF) and diffuse EBER-1 positivity as EBER-positive cells in ≥ 5/HPF (14). Nissen et al. and Zhou et al. divided EBER-positive cases into two groups: cases with low EBV concentrations (< 10/HPF) and high EBV concentrations (≥ 10/HPF) (18, 21). However, a specific cut-off to establish a diagnosis of EBV-related colitis has not been clarified. Accordingly, Shu Xu et al. conducted a study and identified that the best EBER cut-off point for outcome prediction was 2.5/HPF and that the cut-off value for blood EBV DNA was 2035 copies/mL, which was more accurate than the traditional cut-off value of 500 copies/mL, although the sensitivity and specificity were not sufficiently high (22). A prior study by Ciccocioppo et al. suggested a cut-off value of 10^3^ copies/10^5^ cells for EBV DNA in the colonic mucosa to distinguish between superimposed viral colitis and latent viral infection using the highly sensitive RT−qPCR technique (13). These cut-off values may provide better clinician insight for the assessment of the impacts of EBV infection in IBD patients. Although blood testing is a noninvasive and simple method, several studies found a disconnection between the levels of EBV load detected in peripheral blood and that in mucosal samples, suggesting that viral colitis may exist independently of the systemic involvement (12, 13, 18, 21). Moreover, the study by Li et al. identified a slight agreement of PCR and ISH in intestinal mucosa and suggested to using both techniques to detect EBV in clinical practice (20). Further detailed studies are needed to explore the different phases of EBV infection and investigate the best diagnostic methods for EBV-related colitis in IBD patients.

In conclusion, many techniques including PCR, ISH for EBER, and immunohistochemistry have been applied to confirm the presence of EBV infection in the mucosal inflammatory cells of patients with IBD. EBER-ISH is considered as the gold standard to determine EBV infection in intestinal mucosa due to its high sensitivity and precise cellular localization. However, distinguishing between EBV latent infection and superimposed viral colitis remains difficult and the testing of EBER by ISH and EBV-DNA by PCR in intestinal mucosa of IBD seems much more sensitive and accurate than blood testing to some extent.

## The influence of EBV coinfection on the clinical course of IBD

### EBV coinfection may contribute to exacerbation, a poor prognosis and refractory IBD

Prior studies found that the prevalence of EBV in the intestinal mucosa of patients ranged from 16% to 79.4% (10, 11, 13, 14, 19–23). Differences in the enrolled patient populations, regions, means of detection, and study designs may explain the inconsistencies among the results. A recent study by Zhou et al. (21) found that the prevalence of detectable levels of EBV DNA in the blood of patients with IBD was 8.4%, while much higher prevalences of EBV DNA in peripheral blood were found in prior studies [20% in the study by Ciccocioppo et al. (13) and 35% in the study by Magro et al. (26)]. It is worth noting that these studies used different blood samples. Magro et al. (26) used whole blood to detect EBV DNA, while Zhou et al. (21), who reported a much lower prevalence, tested plasma, which may explain the discrepancy in the results. Blood samples used to detect EBV DNA include whole blood, plasma, and mononuclear cells, but plasma is tested more frequently and is more precise for adult patients. However, all these studies concluded that infection with EBV may have a role in the pathogenesis of IBD, exacerbation of symptoms, and the poor clinical course leading to refractory IBD (**Table 1**).

The study by Spieker et al. demonstrated that EBV infection may influence the composition of inflammatory infiltrate in UC and may contribute to self-perpetuation of the disease as well as the development of autoimmune disease (10). Subsequently, the study by Bertalot et al. suggested that EBV infection may be associated with chronic UC (11). A large cross-sectional study comprising 94 patients with IBD identified that EBV genetic material was detected more frequently in patients with IBD than in controls and was detected significantly more frequently in intestinal tissue in patients with disease exacerbation than in patients with remission (12). Moreover, studies by Ciccocioppo et al. revealed a higher prevalence of EBV-DNA in refractory IBD patients than in nonrefractory IBD patients and controls, and the EBV DNA load was positively correlated with the severity of mucosal damage and clinical indexes of activity (13, 16). In addition, they innovatively discovered the potential mechanism of EBV-associated IBD refractory to conventional therapies, in which opportunistic viral particles in inflamed IBD mucosal cells spread from immune cells to epithelial cells, thus resulting in productive viral replication and significantly contributing to tissue damage (16). In a case−control retrospective study using ISH for the detection of EBER1, a much higher proportion of EBER-positive lymphocytes was found in the colectomy specimens of patients with refractory IBD than in controls, and a positive correlation between EBER positivity and the depth of inflammation and mucosal ulceration in patients with refractory IBD was identified (14). Likewise, recent studies formed the same conclusions in cohorts of Chinese patients with IBD (20–23). In addition to these clinical studies, a recent study using an IBD mouse model, namely, the dextran sodium sulfate (DSS) mouse colitis model, detected that the presence of EBV DNA exacerbated colitis by aggravating colonic disease activity and increasing damage to the colon histologic architecture; this result indicates the possibility of potential therapeutic approaches targeting endosomal Toll-like receptor (TLR) signaling, which needs to be tested in large proportions of patients with IBD (27).

The presence of EBV infection in the intestinal mucosa also seems to contribute to the poor prognosis of IBD. A study by Nissen et al. found that high EBV concentrations were more likely to occur in EBV-positive patients undergoing colectomy than in those without colectomy and were associated with the presence of atypical inflammatory infiltration and B-lymphocytes (18). Coinfection with EBV was discovered to be a risk factor for subsequent colectomy in patients with UC in the study by Hosomi et al. (19). A study in a cohort of 92 UC patients by Xu et al. revealed that UC patients with high EBV concentrations had a higher risk of adverse outcomes, including surgery, hospital admission, and a prolonged hospital stay (22). A very recent study aiming to explore the impacts of EBV infection on IBD outcomes also reported that EBV positivity was associated with higher hospital admission and surgery rates and a greater need for therapy escalation (24). However, a study by Zhou et al. found that EBV infection was not associated with a poor prognosis. This discrepancy may result from the small number of patients enrolled since this study included only 27 EBER-1-positive IBD patients (28).

### Possible risk factors for comorbid EBV infection and IBD

Among these studies, age; irregular ulceration; and therapy comprising steroids, biological agents or immunosuppressants were possible risk factors for EBV infection in patients with IBD (13, 18, 21, 22) (**Table 1**). In addition, the study by Zhou et al. found a higher prevalence of EBV infection in UC patients than CD patients and considered UC to be a risk factor for EBER positivity in IBD patients (21). This conclusion is consistent with that of the study by Spieker et al., who discovered higher frequencies of a high EBV load and productive infection in active UC patients than in CD patients and controls, probably related to the characteristics of the TH2 pattern and elevated serum levels of soluble CD30 protein in UC patients (10). Recently, several studies have explored possible mechanisms of the role of EBV infection in the pathogenesis of UC. A study by Wyss et al. indicated that EBV infection may contribute to the severity of colonic inflammation through the EBI2-7α,25-dihydroxycholesterol axis in patients with UC and mice with colitis (29). Another study in 76 UC patients identified that EBV loads were positively correlated with the disease activity of UC and that EBV infection had a potential influence on the decrease in Helios+ FoxP3+ Tregs in severe active UC patients (30).

In conclusion, a high prevalence of EBV infection in the intestinal mucosa is identified in patients with IBD, especially in those who are refractory to traditional therapy. The co-infection of EBV is probably associated with the exacerbation and poor prognosis including surgery rates and more hospital admissions in the clinical course of IBD under the possible risk of age, therapy of steroids, biological agents or immunosuppressants.

## Characteristics of EBV-positive lymphoproliferative diseases in IBD

### Classification of EBV-associated lymphoproliferative diseases

EBV infection is not only associated with the complication of superimposed viral colitis, but can also lead to lymphoplasmacytic infiltration and a heterogeneous spectrum of lymphoproliferative diseases in patients with IBD due to the extensive use of immunomodulators (18, 31–35). According to the 2016 World Health Organization (WHO) classification of lymphoid neoplasms, EBV-associated lymphoproliferative diseases can be divided into the following categories: B-cell lymphoproliferative diseases (B-cell LPDs) and NK/T-cell lymphoproliferative diseases (NK/T-cell LPDs) (**Table 2**) (36, 37). In patients with IBD, cases of EBV-positive mucocutaneous ulcer (EBVMCU), lymphomatoid granulomatosis (LYG), HLH, B-cell lymphoma, and very rare NK/T-cell lymphoma have been reported and well described (**Table 2**) (33, 35, 38–41). Ohshima et al. classified EBV-associated T/NK LPD based on pathological evaluation and molecular data into the following categories: A1 cases, which are polymorphic and polyclonal; A2 cases, which are polymorphic and monoclonal; A3 cases, which are monomorphic and monoclonal; and B cases, which are monomorphic and monoclonal and have a fulminant clinical course (42). This classification applies to EBV-associated lymphoproliferative diseases in patients with IBD and helps both pathologists and clinicians better define and differentiate these diseases. The specific threshold of EBV load to predict the onset of EBV-associated lymphoproliferative diseases has not been determined, but Nissen et al. found that high EBV concentrations with EBER-positive cells in ≥ 10/HPF were significantly more frequent in the monomorphic groups (18); this can provide a reference for clinicians.

**Table 2:** Epstein–Barr virus-associated B-cell or NK/T-cell lymphoproliferative diseases.

Epstein–Barr virus-associated B-cell lymphoproliferative diseases	Epstein–Barr virus-associated NK/T-cell lymphoproliferative diseases
Infectious mononucleosis	EBV-associated hemophagocytic lymphohistiocytosis*
CAEBV of B-cell type	CAEBV-type T/NK-cell disease
Burkitt lymphoma	Systemic chronic active EBV infection of T-cells or NK-cells
Hodgkin lymphoma*	Cutaneous forms of CAEBV
Primary effusion lymphoma	Malignant T/NK-cell disease
HHV8-positive lymphoproliferative disorder	Systemic EBV-positive T-cell lymphoma of childhood and young adulthood
EBV–positive diffuse large B-cell lymphoma, not otherwise specified*	Extranodal NK/T-cell lymphoma, nasal type*
EBVMCU*	Extranasal NK/T-cell lymphoma*
Diffuse large B-cell lymphoma associated with chronic inflammation	Aggressive NK-cell leukemia
Lymphomatoid granulomatosis*	EBV-positive nodal NK/T-cell lymphoma
	Intestinal T-cell lymphoproliferative disease

EBV, Epstein–Barr virus; CAEBV, chronic active Epstein–Barr virus infection; NK, natural killer. *Epstein–Barr virus-associated lymphoproliferative diseases complicated in patients with inflammatory bowel disease.

### EBV-positive mucocutaneous ulcer

EBVMCU, as a new entity recognized in the 2016 review of the WHO classification of lymphoid neoplasms (36), was first described in a study by Dojcinov et al. in 2010 that included 26 patients receiving different types of immunosuppression, including one patient with UC (43). In general, EBVMCU is a very rare B-cell lymphoproliferative disease associated with age-related immunosenescence or iatrogenic immunosuppression. It is morphologically characterized by shallow, sharply circumscribed ulcers involving the oropharyngeal mucosa, skin, or gastrointestinal tract, and it is pathologically characterized by a polymorphous infiltrate of small lymphocytes, histiocytes, plasma cells, eosinophils, and atypical large B-cell blasts, often with Hodgkin/Reed-Sternberg (HRS) cell-like morphology. It is immunologically characterized by a B-cell immunophenotype in the lesional immunoblasts with positive staining of CD30, CD15, CD20, CD79a, CD45, MUM1, PAX5, OCT-2, and BCL-6 and genetically characterized by EBER positivity in the infiltrating cells on ISH and the presence of clonal Ig gene rearrangements and monoclonal or clonally restricted T-cell patterns compatible with the restricted T-cell response against EBV infection on PCR in a subset of cases (43, 44). Very few cases have been reported in patients with IBD who mostly received therapy comprising immunosuppressive agents, including methotrexate (MTX), azathioprine (AZA) or 6-mercaptopurine (6-MP), or combination therapy with anti-TNF drugs, including infliximab, adalimumab or golimumab (31, 40, 44–47), though some cases have possibly been misdiagnosed or are unpublished. The clinical course of EBVMCU is mostly self-limiting and indolent, and the lesions tend to regress spontaneously after the withdrawal of immunosuppressants and respond well to conservative management (40, 43). However, with the increase in the number of cases reported, scientists found that EBVMCU can exhibit an aggressive course and may require aggressive therapy to prevent disease progression (44). Moran NR et al. first reported a case of EBVMCU progressing to widespread Hodgkin lymphoma in a patient with CD who underwent emergency colectomy and subsequent urgent chemotherapy (45). There are also reports of cases that required CD20- and CD30-directed antibody therapy, such as rituximab and brentuximab, to achieve clinical recovery; providing new therapeutic strategies for some severe cases of EBVMCU (31, 47).

### Lymphomatoid granulomatosis

LYG is also a rare EBV-driven B-cell lymphoproliferative disease (LPD) clinically characterized by universal involvement of the lungs as well as other common extranodal sites, including the skin, central nervous system, liver, and kidneys. It is pathologically characterized by infiltrate composed of EBV-positive atypical B cells with different numbers and densities on a graded basis, angioinvasive/angiodestructive reactive T-cell infiltrate, and various degrees of necrosis. Treatment methods for LYG include immune modulation of interferon-α2b in low-grade disease, immunochemotherapy in high-grade disease, and crossover treatment in some cases of relapse or progression (48). LYG rarely occurs in patients with IBD. Subramaniam et al. reported a case of EBV-associated lymphoproliferative disease resembling LYG based on histopathological examination of tissue obtained from a large necrotic gastric ulcer and a necrotic pulmonary nodule subjected to immunochemotherapy in a 42-year-old woman with CD, but this patient suffered from various severe complications and finally died from refractory disease involving the central nervous system (41). Thus, the detailed management of LYG in patients with IBD remains unclear because of the rarity of this disease.

### Hemophagocytic lymphohistiocytosis

Another rare complication, HLH, has been increasingly reported in IBD patients with EBV infection who are exposed to immunosuppressants or biologics (3, 31, 38, 49–52). HLH is a life-threatening clinical syndrome with symptoms of prolonged fever, hepatosplenomegaly and pancytopenia. Characteristic clinical findings include increased ferritin, triglyceride, transaminase, bilirubin, lactate dehydrogenase, and soluble interleukin-2 receptor α-chain levels; decreased fibrinogen levels; characteristic bone marrow aspirate results; increased numbers of macrophages; and evidence of hemophagocytosis (53). Many cases of HLH complicated with IBD have been reported in pediatric patients or adolescents who are more prone to suffer from primary EBV infection (31, 38, 49, 51, 54), and a respective study revealed that pediatric patients with CD and thiopurine administration had a 100-fold higher risk for the development of HLH (39). However, it is worth noting that some cases of HLH occur in adult patients with IBD (3, 50, 52, 55). CD seemed to be a risk factor for the development of HLH among the reported cases; this result was also found in Li et al.’s review, and Thompson et al. suspected that there may be underlying disease-specific factors contributing to HLH due to the importance of the Th1-driven cytokine response in both CD and HLH (52, 56, 57). Very few reports analyzed the function of NK cells. Two case reports identified NK cell deficiency in a teenage patient and an adult patient, respectively, and they were successfully treated with only rituximab therapy, which eliminates EBV-infected B cells and is more specific and less toxic than traditional chemotherapy (51, 52). Thus, early recognition, diagnosis, and treatment of HLH in susceptible IBD patients are of vital importance to prevent disease progression since HLH tends to be associated with a poor outcome, and several cases are complicated by EBV-associated lymphoproliferation and even malignant lymphomas (3, 50, 54). In addition, the diagnosis of X-linked proliferative (XLP) syndrome should also be taken into consideration when HLH occurs in a young patient with IBD associated with primary EBV infection, as reported by Hügle et al. (58).

### EBV-associated lymphomas

An increasing number of EBV-associated lymphoproliferative diseases and lymphomas have been identified as complications of IBD, including Hodgkin lymphoma (34, 41, 59–61), diffuse large B-cell lymphoma, NK/T-cell lymphoma (33, 35, 62–65), and some unclassifiable B-cell lymphoproliferative disorders (3, 66–69). Since the clinical symptoms of intestinal lymphoproliferative diseases are diverse and tend to be similar to those of IBD and the final diagnosis mainly relies on histopathological and immunophenotypic examinations of intestinal biopsy or surgical resection tissue, scientists have recommended examining deeper intestinal tissue to observe atypical infiltration of B or T/NK lymphocytes and the specific immunophenotypes of different lymphomas to avoid misdiagnosis and treatment delay, especially when IBD becomes severe and refractory (34, 63). It is challenging to elucidate the exact role of EBV in the pathogenesis of lymphomas in patients with IBD, and EBV possibly contributes differently to different types of lymphomas. EBV can have an antiapoptotic function, such as c-myc deregulation or loss of B-cell antigen receptor (BCR) function, in B-cell lymphomas, while the mechanism of EBV in NK/T-cell lymphomas remains unknown. Similar to that of in PTLD, the pathogenesis of lymphoma in patients with IBD may be attributed to immunosuppression by immunosuppressive therapy, which impairs the immunosurveillance system and CTL immune activity, reactivates EBV latent infection and promotes oncogenic function, possibly related to the chronic inflammatory microenvironment in IBD (4, 70). Future studies are needed to explore the molecular mechanisms of EBV-associated lymphomas in patients with IBD exposed and unexposed to immunosuppressants to better explain the relationship among EBV, lymphomas and IBD. Although lymphoproliferative diseases in IBD patients share similar clinicopathological characteristics with PTLDs, the prognosis of lymphoproliferative diseases in IBD patients seems to be better than that of PTLD after stopping the use of immunosuppressive therapy and receiving chemotherapy when necessary (18, 71).

### Risk assessment of EBV-associated lymphomas in patients with IBD

It is still unclear whether there is an association between lymphoma and IBD, and prior studies found no significantly increased risk of lymphomas in patients with IBD compared with the general population (72, 73). However, the association between lymphoma and immunosuppressive therapy in patients with IBD has been clarified in many studies (15, 32, 74–78) on the basis of the common use of azathioprine and 6-mercaptopurine for the treatment of steroid-dependent or steroid-refractory IBD (79) (**Table 3**). The study by Dayharsh et al. reported a slight association between immunosuppressant (azathioprine, 6-mercaptopurine) use and EBV-positive lymphoma in patients with IBD (15). The first large prospective study, the CESAME cohort study (32), revealed that patients with IBD receiving thiopurines had a higher risk of development of lymphoproliferative diseases than those who had never used the drugs, and the overall multivariate hazard ratio was 5.28 (95% confidence interval [CI], 2.01-13.9). Although this study did not specify the EBV involvement, most cases of lymphoproliferative diseases in this cohort were post-transplant lymphoproliferative disorder-like diseases of B-cell origin and were associated with EBV infection. This study also suggested that older age, male sex, and a longer course of IBD were additional risk factors for lymphomas in IBD (32), which was in accordance with the results of a case−control study (77). Interestingly, an increased risk of EBV-positive lymphoma was also observed in younger adults (<50 years) with IBD in the study by Vos et al. (75). In this nationwide study involving a cohort of 17,834 IBD patients, 92% of the patients complicated with EBV-positive lymphoma were exposed to azathioprine or 6-mercaptopurine, compared with 19% of the patients with EBV-negative lymphoma, implying a strong association between EBV-positive lymphoma in IBD and thiopurine use (75).

**Table 3:** Risk factors for lymphoproliferative diseases in IBD patients.

Complicated lymphoproliferative diseases in IBD patients	Risk factors	Risk associated with immunosuppressant use	References
EBVMCU	azathioprine use	NA	Matnani et al. (40)
EBVMCU	azathioprine and anti-TNF drug use	NA	Teixeira Mendes et al. (46) Montes et al. (47)
LYG	6-mercaptopurine use	NA	Subramaniam et al. (41)
EBV-related HLH	thiopurine use	NA	Posthuma et al. (38) Serrate et al. (50) N’Guyen et al. (3) Fitzgerald et al. (51) Virdis et al. (54)
EBV-related HLH	infliximab use	NA	Salado et al. (55)
EBV-related HLH	azathioprine and infliximab use	NA	Francolla et al. (49)
EBV-positive lymphomas	azathioprine and 6-mercaptopurine use	slightly increased risk	Dayharsh et al. (15)
lymphomas	azathioprine and 6-mercaptopurine use	RR: 4.18 (95% CI, 2.07-7.51)	Kandiel et al. (74)
lymphoproliferative diseases	thiopurine use, older age, male sex, longer duration of IBD	HR: 5·28 (95% CI, 2.01–13.9)	Beaugerie et al. (32)
NHL	combination anti–TNF and immunomodulator therapy	SIR: 3.23 (95% CI, 1.5-6.9)	Siegel et al. (80)
EBV-positive lymphomas	azathioprine and 6-mercaptopurine use	NA	Vos et al. (75)
lymphoma	thiopurine use alone anti-TNF with thiopurine use	thiopurine alone: SIRR: 1.4 (95% CI, 1.2–2.7) for current use anti-TNF with thiopurine: SIRR: 5.5 (95% CI, 4.5–6.6) for past use and 4.4 (95% CI, 3.4–5.4) for current use	Herrinton et al. (73)
lymphoma	older age, male sex, immunosuppressive medication use	OR: 4.20 (95% CI, 1.35-13.11)	Afif et al. (77)
lymphoma	thiopurine use	HR: 4.2 (95% CI, 2.5–6.8)	Khan et al. (78)
lymphoma	thiopurine monotherapy anti-therapy monotherapy combination therapy	thiopurine monotherapy: aHR: 2.60 (95% CI, 1.96-3.44) anti-TNF monotherapy: aHR: 2.41 (95% CI, 1.60-3.64) combination therapy: aHR: 6.11 (95% CI, 3.46-10.8)	Lemaitre et al. (81)

IBD, inflammatory bowel disease; EBV, Epstein–Barr virus; EBVMCU, EBV-positive mucocutaneous ulcer; HLH, hemophagocytic lymphohistiocytosis; anti-TNF, antitumor necrosis factor; RR, relative risk; HR, hazard risk; aHR, adjusted hazard risk; SIR, standardized incidence ratio; SIRR, standardized incidence rate ratio; NA, not applicable.

The risk of incident lymphoma in patients with IBD receiving anti-TNF therapy remains controversial because few patients receive anti-TNF monotherapy; most of them are treated with thiopurines alone or combined with immunosuppressants and anti-TNF agents (73, 80). Therefore, Lemaitre et al. conducted a nationwide cohort study to assess the risk of lymphoma in adult patients with IBD exposed to thiopurine monotherapy, anti-TNF (infliximab, adalimumab) monotherapy and combination therapy and discovered that thiopurine monotherapy, anti-TNF monotherapy and combination therapy were all associated with a significantly increased risk of lymphoma in IBD patients compared with unexposed patients. Moreover, the risk associated with combination therapy was higher than those associated with thiopurine monotherapy and anti-TNF monotherapy (81). Unlike that for infliximab and adalimumab, which are widely used in IBD patients, no data on the risk associated with therapy with other anti-TNF agents, such as golimumab and certolizumab pegol, in IBD patients complicated with lymphomas have been available. In addition, the association between the use of anti-TNF agents and EBV-associated lymphomas in IBD patients remains unclear because EBV status and the association with EBV infection were not detected in IBD patients with lymphomas in prior studies.

Schwartz et al. suggested that the increased risk of lymphomas in IBD patients was associated with a high dose and long use duration of immunosuppressants, but the exact dose and duration remain unclear (62); additionally, studies found that there tended to be a long duration between the onset of IBD and the occurrence of lymphoma (32, 76). No studies have proven the association between chronic inflammation or disease activity and lymphoma in patients with IBD. In contrast, it has been clearly shown that there is an increased risk of lymphoma in rheumatoid arthritis patients; and this risk is strongly associated with disease activity (82, 83).

In summary, EBV infection predisposes patients with IBD to develop lymphoproliferative diseases, including EBV-positive mucocutaneous ulcer (EBVMCU), lymphomatoid granulomatosis (LYG), hemophagocytic lymphohistiocytosis (HLH), B-cell lymphoma, and very rare NK/T-cell lymphoma which is usually associated with therapy of immunosuppressants or biologics. In addition, old age, male sex, and longer duration of IBD are also associated with increased risk for lymphomas in IBD. The pathogenesis of EBV-associated LDs in IBD has not been clarified and is probably related to the impairment of CTL function caused by immunosuppression.

## Challenges associated with the diagnosis, differential diagnosis and selection of therapy for EBV infection in IBD patients

To date, no international guidelines for the diagnosis of and therapy for complications caused by EBV infection in patients with IBD exist, and many questions remain to be answered and explored in clinical practice.

### Whether and when to screen for EBV infection and monitor EBV infection status

Whether and when to identify EBV infection status are controversial topics. A prospective cohort study in Canada conducted serological testing for VCA-IgM, VCA-IgG, and EBNA-IgG in 263 patients, and the results showed that the prevalence of EBV seronegativity in the IBD population aged 18-25 years was 29%, which was similar to that in the general population; however, EBV seropositivity reached 100% in those older than 25 years, and seropositivity was associated with thiopurine use (84). This supports that younger patients are at increased risk for primary EBV infection, which may result in fatal outcomes such as hemophagocytic lymphohistiocytosis. A recent study by Jennifer Bachmann et al. also confirmed that children with IBD treated with thiopurines had a higher risk of primary EBV infection and developing HLH and suggested to offer functional or genetic testing for XIAP to male patients with EBV-related complications and those with therapy refractory severe CD-like disease during follow-up in a large single center cohort of children with IBD (85). Therefore, EBV testing in younger patients with IBD before the initiation of immunosuppressive drugs is suggested (38). However, the largest study to date on the seroprevalence of EBV infection in adult patients with IBD found that the overall seroprevalence of EBV infection was 97.4%, which suggested the existence of a risk of primary EBV infection in a small percentage of adults with IBD (86). Moreover, a recent multicenter, cross-sectional, observational study in Japan revealed that the average age at the time of primary EBV infection was older than previously reported, with a significant number of uninfected patients in their 20s (87). The latest European Crohn’s and Colitis Organisation (ECCO) guidelines suggest that the use of thiopurines in EBV-IgG-negative patients should be carefully considered (88). Thus, it is of vital importance to screen for EBV infection when considering the administration of immunosuppressants in patients with IBD at any age, not only younger patients. Of course, as discussed above, most adult patients with IBD suffer from complications including superimposed EBV colitis and lymphoproliferative diseases caused by EBV reactivation from latent infection associated with immunosuppressive therapy (22, 32). If the symptoms in IBD patients receiving immunosuppressants become more severe or refractory to traditional drugs or present frequent relapse and endoscopic examination identifies irregular ulcerations, EBV detection in intestinal biopsy or surgical specimens by PCR or EBER-ISH should be considered (14, 22). In addition, considering that the EBER-ISH technique is costly and infrequently performed in clinical practice, it is recommended determining the EBV load in the intestinal mucosa of patients with IBD when the histopathological findings show atypical inflammatory infiltration and/or B-lymphocytes (18). It is also worth noting that opportunistic EBV infection in patents with IBD can develop into malignant lymphoma, and the diagnosis can be a major challenge for clinicians (75). Further studies are needed to explore whether and when to screen for EBV infection and monitor the status of EBV infection and the best testing method to determine the cut-off point of the EBV load to predict the development of EBV-associated complications in patients with IBD.

### Misdiagnosis of IBD and EBV-associated lymphoproliferative diseases

**Figure 2:**
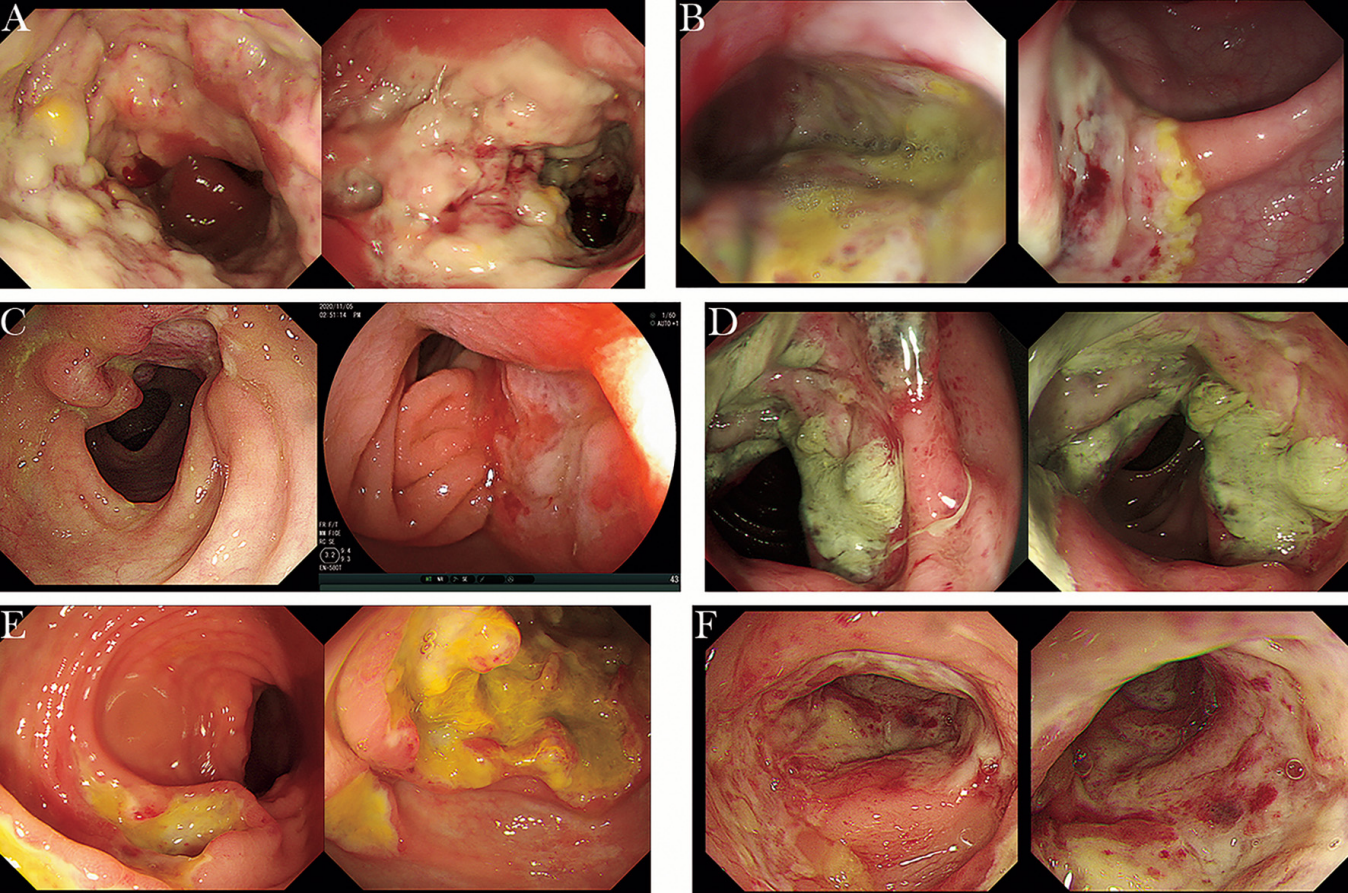
The endoscopic manifestations of intestinal NKTCL (cases from Renji Hospital Affiliated to Shanghai Jiaotong University School of Medicine). **(A)** Extensive and irregular ulcers in the sigmoid colon, accompanied by mucosal hyperplasia and covered with yellow and white adhesive material. **(B)** Multiple large, irregular ulcers covered with white adhesive material in the transverse colon and sigmoid colon. **(C)** A large bulging lesion involving one half of the lumen with a sunken ulcer in the center covered with pus in the ileum and large ulcerative foci in the colon (30 cm from the anus). **(D)** Annular mucosal proliferative lesion with mucosal hyperemia and edema in the transverse colon. **(E)** Circumferential large proliferative lesion in the ileocecum and numerous circular ulcers in the transverse colon. **(F)** Multiple, irregular ulcers with rigid, narrow and congestive lumen in the sigmoid colon.

In the absence of complications caused by EBV infection in IBD patients, it can be difficult to differentiate IBD from rare EBV-associated intestinal lymphoproliferative diseases such as EBVMCU, HLH, systemic CAEBV involving the gastrointestinal tract, and very rare lymphomas, including EBV-positive diffuse large B-cell lymphoma (EBV^+^ DLBCL), nasal type extranodal NK/T-cell lymphoma, intestinal T-cell LPD, Hodgkin lymphoma, Burkitt lymphoma, and extracavitary primary effusion lymphoma (EPEL), because of their overlapping and nonspecific clinical symptoms and endoscopic manifestations (3, 34, 89–92). CAEBV involving the gastrointestinal tract is most commonly misdiagnosed as refractory IBD due to overlapping clinical symptoms, laboratory findings, and endoscopic manifestations. In addition to the common gastrointestinal symptoms, including diarrhea, abdominal pain, and hematochezia, patients with CAEBV often have systemic symptoms, such as intermittent fever, hepatomegaly, splenomegaly, and lymphadenopathy, which are rare in IBD. Other characteristics to help differentiate CAEBV from IBD include extremely high levels of ferritin associated with EBV infection and atypical endoscopic manifestations (91, 93). It is also of vital significance to detect the EBV load by qPCR or EBER-ISH in both peripheral blood and intestinal mucosa because the EBV load in patients with CAEBV is much higher than that in patients with IBD. Although no international criteria for the threshold of EBV load to determine active EBV infection have been established, prior CAEBV cases series found that patients often had more than 105 copies/mL of EBV DNA in peripheral blood, more than 100 EBV+ cells/HPF in surgery samples, and more than 30 EBV+ cells/HPF in biopsy samples, which provides support for clinicians for a correct diagnosis (91, 93). Because CAEBV usually has a poor prognosis and has the potential to progress to hemophagocytic lymphohistiocytosis or malignant NK/T lymphoma, especially when involving T or NK cells in Asian populations, early diagnosis of the disease is important, and the only proven curative treatment for the disease is hematopoietic stem cell transplantation (94, 95). Intestinal lymphoma is a common differential diagnosis for IBD in clinical practice, but misdiagnosis in cases of EBV-positive primary intestinal lymphoma is not uncommon, especially primary EBV-positive intestinal NK/T-cell lymphomas (NKTCLs). The gastrointestinal tract is the most common primary site of extranodal lymphomas, and intestinal NKTCL is a relatively rare type with a high degree of malignancy that most commonly involves the colon and part of the small intestine (96). The symptoms that overlap with IBD include fever, abdominal pain, diarrhea, and complications such as perforation and fistula. However, the intermittent pattern of fever in patients with primary intestinal NKTCL can help differentiate it from IBD. The endoscopic manifestations (**Figure 2**) and pathological findings of primary intestinal NKTCL are atypical and resemble those of IBD. However, primary intestinal NKTCL possesses some distinctive immunophenotypic features, including NK/T cells [CD3 positive] expressing EBER, CD56 [NK-cell type] and monoclonal TCRγ [T-cell type] genes, and negative expression of CD5 (97). Thus, repeat deep endoscopic biopsy combined with examination of the immunophenotype, detection of EBV infection, and exploratory laparotomy should be considered to help establish the early correct diagnosis when intestinal malignant lymphoma is highly suspected (89, 90, 97, 98).

### Controversy regarding the therapeutic schedule for EBV infection in IBD patients

The therapeutic schedule for patients with IBD complicated with EBV infection is controversial. In most cases, a reduction in or withdrawal of immunosuppressants successfully aided in achieving remission of disease when EBV infection exacerbated the severity of inflammation and resulted in atypical inflammatory infiltration or lymphocytes in the intestinal mucosa of patients with IBD (18). However, therapeutic modifications, including immunosuppressant changes at EBV diagnosis, can also be complex in clinical practice (24). Prophylactic antiviral use has been proven to be an effective strategy to reduce the risk of PTLD (99), but controversy remains about antiviral therapy in patients with IBD complicated with EBV infection. Two case reports showed that the addition of ganciclovir or acyclovir helped improve symptoms in patients with IBD and EBV-associated complications (31, 100), while the study by Ciccocioppo et al. indicated that antiviral therapy was ineffective in refractory IBD patients with EBV-related colitis (13). More prospective studies are needed to specify the role of antiviral therapy for EBV infection and explore potential effective therapeutic measures.

## Conclusion

EBV infection is very common in patients with IBD and can cause various complications, including superimposed EBV-related colitis and lymphoproliferative disorders. Thus, it is important to screen for EBV and monitor the status of EBV infection in patients with IBD, especially those who are EBV-seronegative and have a risk of primary EBV infection. Clinicians should recognize the presence of EBV infection to make a correct diagnosis as early as possible and avoid misdiagnosis in patients with refractory IBD.

## Author contributions

HZ (First Author): Conceptualization, Writing-Original Draft; SZ (Corresponding Author): Conceptualization, Funding Acquisition, Supervision, Writing-Review & Editing; ZC (Corresponding Author): Conceptualization, Funding Acquisition, Supervision, Writing-Review & Editing. All authors contributed to the article and approved the submitted version.

## Funding

Establishment and application of inflammatory bowel disease cohort database, No. SHDC2020CR6020, Scientific Research Project of Shanghai Science and Technology Commission, No.22Y11907900 and the National Natural Science Foundation of China (Grant No. 81972655).

## Conflict of interest

The authors declare that the research was conducted in the absence of any commercial or financial relationships that could be construed as a potential conflict of interest.

## Publisher’s note

All claims expressed in this article are solely those of the authors and do not necessarily represent those of their affiliated organizations, or those of the publisher, the editors and the reviewers. Any product that may be evaluated in this article, or claim that may be made by its manufacturer, is not guaranteed or endorsed by the publisher.
